# Sex differences concerning the effects of ankle muscle fatigue on static postural control and spinal proprioceptive input at the ankle

**DOI:** 10.3389/fnhum.2023.1015597

**Published:** 2023-07-05

**Authors:** Donguk Jo, Martin Bilodeau

**Affiliations:** ^1^School of Rehabilitation Sciences, Faculty of Health Sciences, University of Ottawa, Ottawa, ON, Canada; ^2^Aging and Movement Laboratory, Bruyère Research Institute, Ottawa, ON, Canada; ^3^School of Human Kinetics, Faculty of Health Sciences, University of Ottawa, Ottawa, ON, Canada; ^4^LIFE Research Institute, University of Ottawa, Ottawa, ON, Canada

**Keywords:** sex differences, postural control, muscle fatigue, soleus H-reflex, proprioceptive inputs

## Abstract

**Aims:**

The main aim of this study was to determine sex differences in postural control changes with ankle muscle fatigue during a standing forward leaning (FL) task under different vision conditions. The secondary aim was to examine sex differences in the effect of fatigue on soleus (SOL) H-reflex amplitude, a measure of motoneuron excitability with activation of Ia afferents.

**Methods:**

Fifteen healthy young adult males (mean age: 28.0 years) and 16 healthy young adult females (mean age: 26.1 years) were asked to perform four consecutive FL tasks [30 s; two with eyes open (EO) and two with eyes closed (EC)] before, and immediately following a fatiguing exercise consisting of alternating ankle plantarflexion (6 s) and dorsiflexion (2 s) maximal isometric contractions, and at 5 and 10 min of recovery. Center of pressure (COP) sway variables (mean position, standard deviation, ellipse area, average velocity, and frequency), an ankle co-contraction index, and a ratio of SOL H-reflex to the maximum amplitude of the compound muscle action potential (M-max) were obtained during the FL tasks. A rating of perceived fatigue (RPF) was also documented at the different time points.

**Results:**

Time to task failure (reduction of 50% in maximal voluntary isometric contraction torque of ankle plantar flexors) and the increase in RPF value were not significantly different between males and females. Both sex groups showed similar and significant increases (*p* < 0.05) in mean COP sway velocity with no significant changes in co-contraction indices. No significant effects of fatigue and related interactions were found for SOL H/M-max ratio.

**Discussion:**

The absence of a significant sex difference in postural control change (sway and co-contraction) with fatigue could be explained by similar perceived (RPF) and performance fatigability (exercise duration) between males and females in the present study. Fatigue did not lead to significant changes in SOL spinal motoneuron excitability with activation of Ia afferents.

## 1. Introduction

Postural stability is regarded as the capability to control the center of mass with respect to the base of support (Shumway-Cook and Woollacott, 2012). To maintain postural stability, the central nervous system must rapidly integrate sensory information from the visual, vestibular, and somatosensory systems and use this information selectively to produce complex motor responses (i.e., timing, direction, and magnitude), appropriate to the characteristics of the postural stability disturbances associated with a given task and of the surrounding environment (Maki and McIlroy, 1996).

Exercise-induced fatigue of postural muscles (trunk, hip, knee, and ankle) can alter or impair the control of postural stability, mainly due to fatigue-induced modifications in the peripheral proprioceptive system and central processing of sensory inputs, as well as decrements in force production capacity (Enoka et al., 2011; Paillard, 2012; Papa et al., 2015). In particular, ankle muscle fatigue can lead to significant postural control alterations during tasks such as upright standing on one or two legs (Bisson et al., 2010, 2011, 2014; Boyas et al., 2011). This effect could involve a change in ankle stiffness through increased coactivation of antagonists, given the significant role of ankle muscles in regulating active ankle stiffness to maintain postural stability (Loram and Lakie, 2002; Di Giulio et al., 2009). For example, several studies (Boyas et al., 2013a,b,c) found increases in COP sway velocity and/or frequency with ankle muscle fatigue during static tasks, suggesting an increase in ankle stiffness to maintain stability optimally in a state of fatigue. Also, the absence of vision may increase the extent of such fatigue-related changes in postural control during relatively challenging static standing tasks. This has been observed in a few studies (Vuillerme et al., 2001; Soleimanifar et al., 2012; Boyas et al., 2013a) where fatigue of ankle plantar flexors (PF) led to greater impairments in postural stability during one-leg standing with eyes closed (EC) compared with eyes open (EO).

Given such significant effects of muscle fatigue on postural control, sex differences in muscle fatigue may lead to different postural control changes between males and females. It has been suggested that females are generally less fatigable than males during isometric fatiguing exercise (Hunter, 2014, 2016), mainly due to: a greater proportion of fatigue-resistant muscle fibers within skeletal muscles (Hunter, 2016), lesser neural drive to the muscle (Russ and Kent-Braun, 2003), and greater oxidative metabolic capacity (Russ et al., 2005) in the former group. This sex difference in muscle fatigue may lead to greater postural control changes with fatigue in males compared with females. For example, greater center of pressure (COP) sway displacements (Springer and Pincivero, 2009), increased COP velocity (only in males) during one-leg standing (Bannon et al., 2018), and reduced leg-reaching distance (Gribble et al., 2009) have been found in males compared with females in a state of fatigue of ankle, knee, and hip muscles. These findings may be due to different postural control strategies used in the presence of fatigue between males and females. In particular, an ankle strategy (ankle joint control or movements) to maintain stability could be a primary contributor to such sex differences (e.g., during quiet standing, see Wojcik et al., 2011). However, sex differences in postural control changes with ankle muscle fatigue have only been sparsely studied, including in challenging tasks, such as when leaning forward close to the limit of stability (LOS).

Furthermore, ankle muscle fatigue could lead to a degradation in the information provided by proprioceptive afferents from the fatigued muscles, which could contribute to fatigue-induced changes in postural sway or performance (Paillard, 2012). Variation in proprioceptive inputs from the ankle can be partly reflected by modulation of the soleus (SOL) H-reflex, an electrically evoked response analogous to the stretch reflex, but which bypasses muscle spindles (Zehr, 2002; Palmieri et al., 2004). The H-reflex is considered an index of the efficacy of the transmission between muscle spindle afferents (Ia afferents) and alpha motor neurons (spinal pathway) (Baudry, 2016). In the context of muscle fatigue and postural control, two studies at this moment have reported a decrease in SOL H-reflex amplitude following repetitive standing tiptoes (Laudani et al., 2009) or jumps (Ritzmann et al., 2016) in healthy young adults, which was accompanied by an increase or decrease in anteroposterior (AP) COP displacement during upright standing on a firm or movable surface. It was suggested that such SOL H-reflex depression (accompanying fatigue-induced postural changes) may be attributed to reciprocal inhibition due to an increased level of activation in the antagonists [e.g., tibialis anterior (TA)] to compensate for instability with fatigue (Ritzmann et al., 2016). The withdrawal of vision may also lead to a decrease in the SOL H-reflex through increased presynaptic inhibition of Ia afferents (e.g., see Baudry and Duchateau, 2012). However, no studies have looked at SOL H-reflex modulation with fatigue in the context of postural control and potential differences based on sex.

The main aim of this study was to determine sex differences In postural control changes (COP sway parameters and co-contraction) with ankle muscle fatigue during a standing forward leaning (FL) task in different vision conditions (EO vs. EC). A secondary aim was to determine fatigue-induced changes in SOL H-reflex according to sex and vision. It was hypothesized that males would show greater increases in both COP sway parameters and ankle stiffness (co-contraction) compared with females, and such results would be more pronounced with the withdrawal of vision. Also, we hypothesized that ankle muscle fatigue would lead to SOL H-reflex depression, and this depression would be more pronounced in males and with the removal of vision.

## 2. Materials and methods

### 2.1. Participants

General information about participants is shown in Table 1. Fifteen healthy young males (mean of 28.0 ± 4.2 years and range between 22 and 36 years) and 16 healthy young females (mean of 26.1 ± 4.8 years and range between 22 and 37 years; no significant age difference between sexes) volunteered for this study. The participants had no disorders and musculoskeletal injuries. Both sex groups showed a similar moderate-to-high physical activity level (overall: 8.8 ± 1.5 out of 15; males: 8.7 ± 1.6; females: 8.9 ± 1.4), as reported on a habitual physical activity questionnaire documenting work, sport, and leisure time (Baecke et al., 1982). The study was approved by the Research Ethics Boards of the University of Ottawa (# H11-14-23) and Bruyère Continuing Care (#M16-08-038). Participants were informed of all procedures and consented orally before enrolling in the study.

**TABLE 1:** Sex differences in demographics and MVIC torque of ankle muscles.

	Mean (SD)			*p*-Values
	**Overall (*n* = 31)**	**Males (*n* = 15)**	**Females (*n* = 16)**	
Age (years)	27.3 (4.5)	28.0 (4.2)	26.1 (4.8)	0.383
Height (cm)	171.4 (9.7)	178.6 (7.3)	164.6 (6.1)	**<0.001**
Weight (kg)	66.2 (12.9)	74.0 (9.5)	59.0 (11.6)	**<0.001**
PF torque (Nm)	195.8 (62.6)	234.2 (57.0)	159.8 (44.3)	**<0.001**
DF torque (Nm)	64.8 (20.7)	82.69 (13.0)	48.1 (9.0)	**<0.001**

MVIC, maximal voluntary isometric contraction; PF, ankle plantar flexors; DF, ankle dorsiflexors. Bold *p*-values (<0.001) indicate significant differences between males and females.

### 2.2. Experimental procedure

**FIGURE 1:**
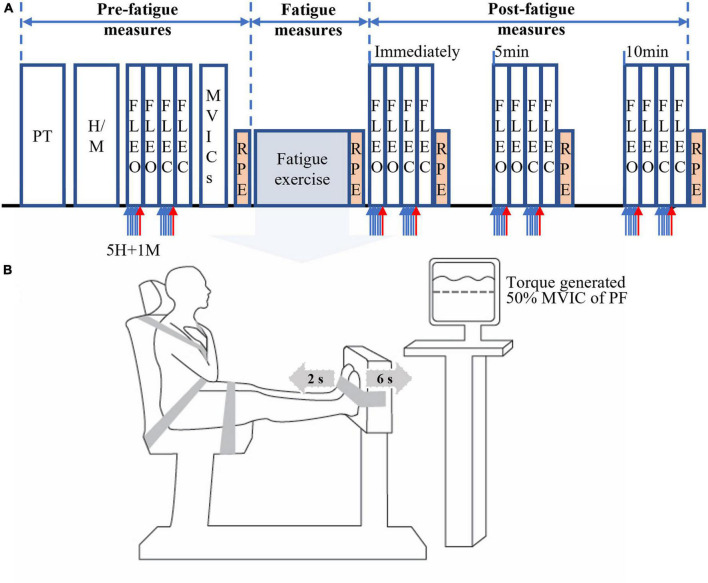
Schematics of the experimental protocol. The schematics include experimental procedures **(A)** and alternating isometric contractions of ankle plantar (for 6 s) and dorsiflexors (for 2 s) on a dynamometer **(B)**. PT, practice of standing forward lean (FL) tasks with eyes open (EO) and eyes closed (EC); H/M, determination of electrical stimuli intensities for obtaining submaximal H-reflex and maximum M-wave (M-max); FLEO/FLEC, the FL task with eyes open and closed; 5H (5 blue arrows), five submaximal electrical stimuli for five H-reflexes; 1M (red arrow), supramaximal stimulus for one M-max; MVICs, maximal voluntary isometric contractions; RPF, rating of perceived fatigue using a 12-point modified Borg’s CR-10 scale. The order of the FL tasks has been counterbalanced.

A schematic of the experimental procedure is shown in Figure 1A. All participants were first asked to practice each of two task conditions (FL-EO and FL-EC) twice. Then, two different levels of electrical stimulus intensity were determined to elicit the maximum amplitude (M-max) of the compound muscle action potential (M-wave) and submaximal SOL H-reflex during FL-EO (see section “2.4. Soleus H-reflex measurements”). Participants were then required to perform four consecutive 30-s FL trials (two FL-EO and two FL-EC) on a force platform. During the first trial of each pair (two FL-EO or two FL-EC), six consecutive electrical stimuli with at least a 3-s interval [as recommended to prevent post-activation depression of the H-reflex (Zehr, 2002)] were delivered to obtain five H-reflexes followed by one M-max. The second trial of each pair did not include the electrical stimuli and was recorded for the assessment of postural stability. All participants then performed a minimum of three bilateral maximal voluntary isometric contractions (MVICs) with ankle PF followed by dorsiflexors (DF), with a 2-min interval between each contraction. The MVICs were maintained over approximately 3 s after the presence of a plateau of the PF or DF torque amplitudes. If the torque was still increasing after the initial three MVICs, then additional trials were performed until no further increase was observed (with a maximum of 5 MVICs; the majority of participants reached their maximum within the first three trials). The highest torque from PF MVICs was used to set a cut-off target (50% MVIC of PF) for an alternating PF-DF isometric fatiguing exercise (see section “2.6. Fatiguing exercise”). Before performing the MVICs, the participants were asked to perform two to three brief sub MVICs of PF and DF, in order to warm up the ankle muscles and familiarize performing the MVICs. All the MVICs were performed in the same position used for the fatiguing exercise (see section “2.6. Fatiguing exercise”). After the fatiguing exercise, the four FL tasks were immediately assessed and also at 5 and 10 min of recovery. The order of the postural tasks (EO or EC) was counterbalanced across participants.

The rating of perceived fatigue (RPF) of the ankle muscles was also assessed using a 12-point modified Borg’s CR-10 scale (0 = “No fatigue at all” to 10 = “Absolutely exhausted”) (Whittaker et al., 2019) before and immediately after the fatiguing exercise and also after each block of four FL trials (immediately, 5 and 10-min post-exercise) (Figure 1A). The obtained RPF values, which was considered perceived fatigue in the present study, were used to indirectly assess the degree of ankle muscle fatigue, as an increase in such RPF value has been significantly correlated with muscle fatigue [e.g., reduction in muscle force or torque (Whittaker et al., 2019) and increased muscle activation (Cruz-Montecinos et al., 2019)].

### 2.3. Electromyographic signal and center of pressure data recordings

Electromyographic (EMG) signals from SOL, gastrocnemius lateralis (GL), and TA of the dominant leg were recorded using bipolar surface electrodes (width: 1 mm, length: 10 mm, and center-to-center interelectrode distance: 10 mm; DE-2.1, Delsys Inc., Boston, MA, USA). The recording electrodes were placed according to the SENIAM recommendations (Hermens et al., 1999). The reference electrode was placed on the patella or the upper part of the tibia of the same leg. These EMG signals were amplified (×1,000; common mode rejection ratio: 92 dB; channel frequency response: 20–450 Hz) and recorded at a sampling rate of 5,000 Hz during the entire duration (30 s) of each FL task. During the same period, COP data (see section “2.7. Data analysis”) were recorded using an AMTI AcuGait force platform (Watertown, MA, USA) and the Balance Trainer software (version 1.05.02) at a 25 Hz sampling rate.

### 2.4. Soleus H-reflex measurements

**FIGURE 2:**
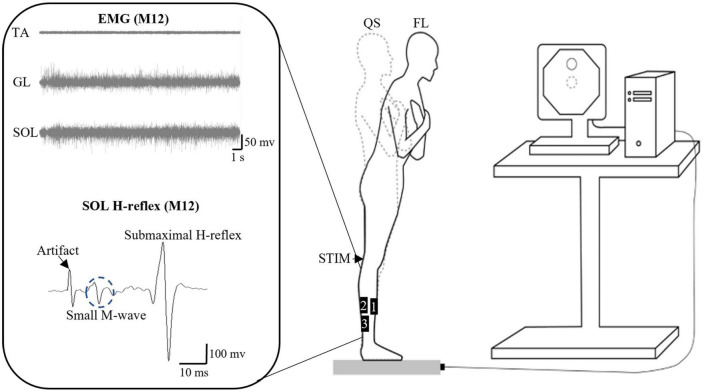
Schematics of the experimental setup. The setup is for the standing forward leaning (FL) task with an example of electromyographic (EMG) signals from tibialis anterior (TA), gastrocnemius lateralis (GL), and soleus (SOL) during the FL task, as well as of SOL H-reflex for participant M12. The FL tasks with eyes open and closed were performed on a force platform with the representation of the COP on a computer screen. EMG data were obtained from the bipolar surface electrodes positioned as follows: 1: TA, 2: GL, and 3: SOL (for H-reflex as well). QS, quiet standing; STIM, electrical stimulation on popliteal fossa for obtaining SOL H-reflex and M-wave.

Soleus H-reflex and M-wave recruitment curves were first obtained when participants with the arms crossed on the chest were standing as still as possible with feet together and staring at a target on a computer screen in front of them. Two stimulating circular electrodes (diameter: 8 mm) were placed longitudinally along the length of the posterior tibial nerve under the dominant knee. Electrical stimuli (pulse duration = 0.2 ms) (Chen and Zhou, 2011) starting from 10 mA were evoked with 10-mA increments up to the presence of a plateau in the SOL M-wave to determine its maximum amplitude (M-max). A supramaximal intensity (10-mA above the intensity needed to reach M-max) was used thereafter to elicit M-max. Subsequently, a 1-mA increment was used starting back from 10 mA for the characterization of the SOL H-reflex recruitment curve up to the point where H-reflex amplitude started to decrease after observing the H-reflex maximum. From the H-reflex recruitment curve, the intensity of electrical stimulation to elicit the test H-reflex during the FL trials was set to meet the following criteria for each participant (Figure 2): (1) H-reflex accompanied by a small M-wave [less than 8% of M-max prior to the H-reflex (Masu and Muramatsu, 2015; Miranda et al., 2019)] to ensure constant stimulus intensity; and (2) placed on the rising portion of the H-reflex recruitment curve to be susceptible to facilitation and inhibition under a given intervention condition (e.g., pre vs. post-fatigue) (Palmieri et al., 2004). The intensity of the electrical stimuli used to elicit the test H-reflex was sometimes slightly re-adjusted during an experiment to ensure that the amplitude of the accompanying small M-wave remained within a ∼2% range for a given participant (intra-subject variation), as suggested by Miranda et al. (2019). These criteria could not be met in six males and seven females, whose data was thus excluded from further analysis (the subset of valid H-reflex data thus included nine males and nine females).

To characterize the potential influence of pre-stimulus muscle activation on H-reflex amplitude, the root mean square EMG amplitude of the ipsilateral antagonist (TA) and agonist muscles (GL and SOL) was calculated over a 50-ms window before the onset of the H-reflex. This was done in order to determine whether reciprocal inhibition (from antagonist activation) and AP postural sway position [which was reported to interact with agonist activation during quiet standing (Tokuno et al., 2007)] affect SOL H-reflex. An increase in TA activation could suggest an increase in reciprocal inhibition from the ankle that contributes to SOL H-reflex depression (Zehr, 2002). A posterior to anterior postural sway shift during quiet standing was reported to lead to a slight increase in the H/M ratio (as well as activation of ankle PF) (Tokuno et al., 2007).

### 2.5. Postural tasks

A schematic of the experimental setup for the FL task is shown in Figure 2. The task was performed using the Balance Trainer software (AMTI, Watertown, MA, USA). The LOS was first obtained by measuring the maximum excursion of the COP while participants lean the whole body in a standing position (feet together) as far as possible forward, backward, and side-to-side on a force platform without lifting the heels or toes and limiting the movement to the ankle. Afterward, participants were asked to lean forward and reach a target set at 70% of the anterior LOS with a representation of their COP on a computer screen placed 1.8 m in front of them. They were required to maintain the 70% LOS target as closely as possible for 30 s with EO or EC. For the EC condition, participants first reached the 70% of anterior LOS target with EO, and then closed their eyes where investigators asked them to memorize the reaching 70% target and to maintain it as close as possible before closing their eyes to start the task. Non-visual feedback or cues were not given during the no-vision task to determine pure vision effects. For both EO and EC conditions, the participant indicated when they felt stable by saying “OK” out loud, which triggered the researcher to begin the 30-s recording.

### 2.6. Fatiguing exercise

A schematic of the experimental setup for the fatiguing exercise is shown in Figure 1B. Participants sat on the chair of a Biodex system 3 Pro (Biodex Medical Systems, Shirley, NY, USA) with their hip at about an 80° angle, legs extended forward and both feet placed on a footplate with the ankle at a 90° (aligning the external malleoli with the center of rotation of the dynamometer). Their trunk, pelvis, and feet were secured with non-elastic straps. Because fatigue of both PF and DF was reported to have greater impact on postural control than fatigue of each muscle group separately (Boyas et al., 2011), an alternating PF-DF isometric fatiguing exercise of both legs was performed in this study. A pilot test was conducted to determine the intensity and duration of contractions that generated simultaneous fatigue of both PF and DF to less than 50% of the pre-fatigue MVIC torque. Based on the pilot testing results and previous work (Kennedy et al., 2011), participants were asked to alternate between maximal isometric contractions of PF (for 6 s) and DF (for 2 s) for the fatigue exercise protocol. The fatigue protocol was stopped when PF torque was reduced to less than 50% of the pre-fatigue MVIC torque for three consecutive seconds. Only the investigators had knowledge of the 50% target torque (displayed on a computer screen), as such target could lead to participants not producing a maximum effort during the fatiguing exercise (i.e., early termination). Verbal encouragement was given throughout.

### 2.7. Data analysis

The following COP variables were obtained from the Balance Trainer Software: mean AP and mediolateral (ML) COP position (cm; average AP and ML displacement from the platform center = $\frac{1}{n}\times \sum_{i=1}^{n}x_{i}$ and $\frac{1}{n}\times \sum_{i=1}^{n}y_{i}$, respectively), standard deviation (SD) of the AP/ML COP position (cm; $x_{S\,D}=\frac{1}{n}\times \sqrt{\sum_{i=1}^{n}{\left(x_{i}-x_{C\,O\,P\,d\,i\,s\,p\,l\,a\,c\,e\,m\,e\,n\,t}\right)}^{2}}$ and $y_{S\,D}=\frac{1}{n}\times \sqrt{\sum_{i=1}^{n}{\left(y_{i}-y_{C\,O\,P\,d\,i\,s\,p\,l\,a\,c\,e\,m\,e\,n\,t}\right)}^{2}}$, respectively), mean COP sway velocity (cm/s; path length per unit time = $\frac{l_{p\,a\,t\,h}}{t}$), and the area of the 95% confidence ellipse of COP sway [cm^2^; area of the 95th percentile ellipse = π × $\sqrt{F\times (x_{s\,d}^{2}+y_{s\,d}^{2}+D})\times \sqrt{F\times (x_{s\,d}^{2}+y_{s\,d}^{2}-D})$]: $l_{p\,a\,t\,h}=\sum_{i=2}^{n}\sqrt{{\left(x_{i}-x_{i-1}\right)}^{2}+{\left(y_{i}-y_{i-1}\right)}^{2}}$; D = $\sqrt{{\left(x_{s\,d}^{2}+y_{s\,d}^{2}\right)}^{2}-4\times \left(x_{s\,d}^{2}\times y_{s\,d}^{2}\right)-\sigma_{x\,y}}.$ *x*_*i*_ or *y*_*i*_ = relative COP position in the ML and AP plane, respectively; *n* = number of data points for a given trial; *F* statistics = 3.00; and *D* = an intermediate value calculated from SD of AP/ML COP position.

Frequency analysis of COP movements was also performed. The power spectrum was obtained via the Discrete Fourier Transform (DFT) feature of the Balance Trainer software for each 30 s trial, and power was analyzed in three frequency bands (0–0.3, 0.3–1, and 1–3 Hz) for both the AP and ML directions. These frequency bands have been suggested to reflect contributions from the visual, vestibular/somatosensory, and proprioceptive systems, respectively (Kanekar et al., 2014). The power in each band was normalized to the total power in the three frequency bands (Kanekar et al., 2014).

In addition, SOL H-reflex peak-to-peak amplitude was normalized to the SOL M-max to form the H/M ratio. The amplitude (root mean square) of TA, GL, and SOL EMG for a 50-ms window prior to the onset of the H-reflex was calculated. The level of co-contraction between TA and GL and between TA and SOL of the dominant leg during the FL tasks without electrical stimuli was also quantified using the formula described by Rudolph et al. (2001): co-contraction index = level of activity of the less active muscle (root mean square of EMG signal amplitude calculated over the 30-s window comprising a given FL task) / level of activity of the more active muscle(s) × sum of the activity of both muscles.

### 2.8. Statistical analysis

Using G* power (version 3.1.9.4), the minimum sample size required was calculated to be a total of at least 6 participants per group to determine significant effects of fatigue, sex, and their interactions using the *F* test (ANOVA: repeated measures, within-between interaction), with a power of 0.95, an effect size of 0.50, and an alpha of 0.05. The effect size was estimated from existing data, with respect to SOL H-reflex changes produced by ankle PF fatigue during upright standing (Laudani et al., 2009).

Independent *t*-tests were used to compare demographic information (age, height, and weight) and MVIC torque of PF and DF between males and females. Separate three-way ANOVAs with one between-subject factor and two within-subjects factors were used to assess whether fatigue [pre-fatigue vs. post-fatigue (immediately, 5, and 10 min)], sex (males vs. females), vision (EO vs. EC), and their interactions were significant for (1) COP variables (mean AP and ML positions, SDs of the AP and ML positions, sway velocity, sway area, and relative power of the three frequency bands for both AP and ML), (2) co-contraction indices (TA-GL and TA-SOL), and (3) the H/M ratio (only including pre and immediately post fatigue data) during the FL task.

In order to assess the potential influence of anthropometric variables on the changes in COP variables with fatigue, the ANOVAs were performed and compared with three types of COP data: (1) non-normalized, and normalized by (2) height, and (3) weight. Two-way ANOVAs were also conducted to assess the effects of fatigue, sex, and their interaction for the RPF value. If Mauchly’s test of Sphericity (within-subjects effect) was significant (≤0.05), the Greenhouse–Geisser correction was used to determine significance. When significance was detected with ANOVA, pairwise comparisons or independent *t*-tests with a Bonferroni correction were performed.

Pearson’s correlation analyses were conducted to assess the relationship between percent change (from pre to immediately post-fatigue) in COP sway variables and co-contraction indices (TA-GL and TA-SOL) during each of the FL tasks (FL-EO and FL-EC). Correlation analyses were also performed to assess the association between H/M ratio and EMG amplitude of each of GL, and SOL (for 50 ms prior to SOL H-reflex) during each of the FL tasks at pre or post-exercise, and between pre to immediately post-fatigue changes in the H/M ratio and RPF value or EMG amplitude of TA (for 50 ms prior to SOL H-reflex) during each of FL-EO and FL-EC.

## 3. Results

Supplementary Appendices A, B show overall results (ANOVAs) for all dependent variables (COP, co-contraction index, H-reflex, and RPF).

### 3.1. Baseline information and measures

Body height, weight, and MVIC torques of PF and DF were found to be significantly greater in males compared with females (Table 1).

### 3.2. Fatiguing exercise duration and RPF

**FIGURE 3:**
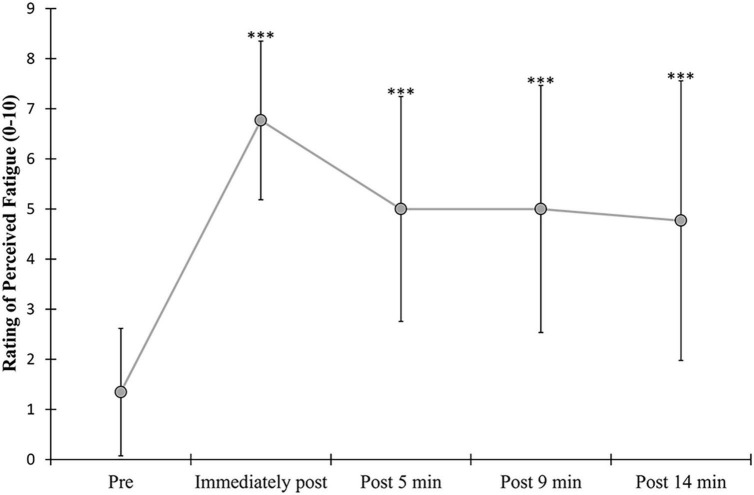
Changes in the rating of perceived fatigue (RPF). RPF scores shown pre-exercise and immediately following alternate isometric exercise of ankle plantar and dorsiflexors, and at the end of each of the four sets of four consecutive standing forward leaning tasks (approximately at 5, 9, and 14 min of recovery). The symbol *** indicates *p* = <0.001. Overall data include standard deviation bars.

Exercise duration, which is the average time needed to reach the 50% decrement in MVIC torque of PF (overall mean = 53.5 ±5.1%, females = 53.5 ± 4.4%, males = 53.5 ±5.9%), was 226.4 ± 201.1 s and was not significantly different (*p* = 0.496) between males (200.4 ± 182.9 s) and females (250 ± 219.8 s). A similar reduction of DF MVIC torque (overall mean = 58.7 ± 11.9%, males = 56.9 ± 12.0%, females = 59.9 ± 12.0%) was observed at the end of exercise. A significant fatigue effect (*F* = 41.37, *p* = <0.001, η^2^ = 0.633) was found for RPF with an expected increase in RPF value up to approximately 14 min following exercise (end of the last four postural tasks) (Figure 3 and Table 2). There was no significant fatigue × sex interaction for RPF.

**TABLE 2:** Mean values (SD) of the rating of perceived fatigue (RPF).

	Mean values (SD) of RPF				
	**Pre**	**Post 0**	**Post 5**	**Post 9**	**Post 14**
Overall	1.346 (1.27)	6.769 (1.58)	5.000 (2.24)	5.000 (2.47)	4.769 (2.79)
Males	1.143 (1.15)	6.500 (1.61)	5.286 (2.16)	5.000 (2.45)	4.786 (2.81)
Females	1.583 (1.41)	7.083 (1.56)	4.667 (2.39)	5.000 (2.59)	4.750 (2.90)

RPF of the ankle muscles was assessed using a 12-point modified Borg’s CR-10 scale (0 = “No fatigue at all” to 10 = “Absolutely exhausted”) before (Pre) and immediately (Post 0) after the fatiguing exercise and also after (Post 4, 9, and 14) each block of four FL trials (immediately, 5 and 10-min post-exercise).

### 3.3. COP sway variables

Statistical results (three-way ANOVAs) for non-normalized COP sway parameters (Supplementary Appendix A) were not different from those with the COP data normalized by height or weight (Supplementary Appendix B), with respect to fatigue effects and related interactions. Results are therefore reported only for the non-normalized data.

#### 3.3.1. Mean COP positions

Only a main effect of vision was significant for mean AP position (*F* = 16.04, *p* = <0.001, η^2^ = 0.356), with an overall slightly more forward position with EO compared with EC (Table 3). For ML mean position, a sex × time (*F* = 3.331, *p* = 0.023, η^2^ = 0.103) interaction were found to be significant, but none of the *post-hoc* t-tests reached significance when comparing pre- to post-exercise percent changes in the ML position between males and females during FL-EO or FL-EC. A sex × vision × time (*F* = 3.293, *p* = 0.024, η^2^ = 0.102) interaction was also found due to a significant left shift of the mean COP position during recovery (immediately to 5 min post-exercise; *p* = 0.02) in males but not in females and only during FL-EC (Table 3).

**TABLE 3:** Mean values (SD) of COP variables.

	COP variables	Mean values (SD)							
		**Eyes open**				**Eyes closed**			
		**Pre**	**Post 0**	**Post 5**	**Post 10**	**Pre**	**Post 0**	**Post 5**	**Post 10**
Overall (*n* = 24)	AP position (cm)^†^	8.316 (1.26)	8.303 (1.26)	8.279 (1.24)	8.292 (1.26)	7.915 (1.25)	8.032 (1.21)	7.775 (1.43)	7.728 (1.51)
	AP SD (cm)^†^	0.366 (0.10)	0.375 (0.08)	0.373 (0.09)	0.377 (0.16)	0.725 (0.25)	0.784 (0.21)	0.766 (0.27)	0.776 (0.27)
	ML position (cm)^¶^	−0.014 (0.68)	−0.017 (0.69)	−0.042 (0.71)	−0.040 (0.14)	0.072 (0.75)	0.240 (0.51)	0.111 (0.78)	0.086 (0.67)
	ML SD (cm)^†^	0.394 (0.10)	0.408 (0.10)	0.406 (0.11)	0.415 (0.12)	0.725 (0.21)	0.725 (0.23)	0.723 (0.24)	0.718 (0.26)
	Sway velocity (cm/s)^†*^	2.163 (0.40)	2.470 (0.56)	2.443 (0.55)	2.408 (0.51)	3.797 (1.42)	4.376 (1.61)	4.012 (1.31)	4.004 (1.53)
	Sway area (cm^2^)^†^	2.798 (1.54)	2.979 (1.49)	2.946 (1.39)	3.046 (1.68)	10.44 (6.56)	11.50 (7.79)	11.24 (7.98)	11.45 (8.84)
Males (*n* = 10)	AP position	8.105 (1.25)	8.121 (1.27)	8.082 (1.26)	8.067 (1.25)	7.584 (1.09)	7.561 (1.03)	7.381 (1.49)	7.189 (1.69)
	AP SD^§^	0.408 (0.12)	0.434 (0.12)	0.414 (0.08)	0.394 (0.08)	0.773 (0.33)	0.891 (0.33)	0.832 (0.26)	0.895 (0.35)
	ML position	−0.157 (0.63)	−0.153 (0.69)	−0.193 (0.68)	−0.182 (0.71)	−0.008 (0.70)	0.221 (0.55)	−0.240 (0.84)	−0.029 (0.76)
	ML SD	0.430 (0.11)	0.437 (0.09)	0.425 (0.10)	0.444 (0.11)	0.735 (0.21)	0.813 (0.26)	0.789 (0.24)	0.791 (0.28)
	Sway velocity	2.249 (0.36)	2.615 (0.51)	2.556 (0.47)	2.510 (0.39)	3.978 (1.72)	4.694 (1.84)	4.286 (1.06)	4.415 (1.62)
	Sway area	3.405 (1.80)	3.657 (1.67)	3.349 (1.31)	3.337 (1.31)	11.28 (7.58)	14.55 (9.52)	13.08 (7.87)	14.34 (10.46)
Females (*n* = 14)	AP position	8.514 (1.27)	8.474 (1.25)	8.464 (1.22)	8.503 (1.27)	8.225 (1.34)	8.475 (1.22)	8.120 (1.32)	8.200 (1.19)
	AP SD	0.326 (0.10)	0.321 (0.08)	0.336 (0.09)	0.361 (0.16)	0.681 (0.25)	0.684 (0.21)	0.709 (0.27)	0.671 (0.27)
	ML position	0.120 (0.72)	0.111 (0.70)	0.100 (0.72)	0.093 (0.72)	0.148 (0.81)	0.258 (0.49)	0.419 (0.59)	0.186 (0.59)
	ML SD	0.359 (0.08)	0.381 (0.10)	0.389 (0.11)	0.388 (0.12)	0.715 (0.21)	0.643 (0.17)	0.665 (0.24)	0.655 (0.25)
	Sway velocity	2.083 (0.44)	2.335 (0.58)	2.337 (0.61)	2.312 (0.60)	3.628 (1.09)	4.077 (1.35)	3.773 (1.49)	3.645 (1.40)
	Sway area	2.230 (1.01)	2.343 (0.98)	2.569 (1.40)	2.774 (1.96)	9.656 (5.59)	8.638 (4.35)	9.626 (7.96)	8.922 (6.45)

These values are assessed before (Pre) and immediately following fatiguing exercise (Post 0), and throughout recovery (5 and 10 min). AP, anteroposterior; SD, standard deviation; ML, mediolateral. ^†^Main effect of vision, values eyes open are significantly lower than with eyes closed (*p* < 0.001). ^§^ Main effect of sex, values in males are significantly greater than in females (*p* < 0.05). *Main effect of time, a value during Pre (before exercise) is significantly greater than during Post 5 and 10 (5 and 10 min after exercise) (*p* < 0.001). ^¶^ Sex × time × vision interaction, only males have a significant left shift from Post 0 (immediately after exercise) to Post 5 during standing forward leaning task only with EC.

#### 3.3.2. SDs of AP and ML COP positions

A main effect of vision was found for both AP SD (*F* = 114.0, *p* = <0.001, η^2^ = 0.797) and ML SD (*F* = 120.2, *p* = <0.001, η^2^ = 0.806), with greater values observed with EC than with EO (Table 3). A significant sex effect was also found for AP SD (*F* = 4.334, *p* = 0.046, η^2^ = 0.103), with males presenting higher values compared with females (Table 3). However, no effects of fatigue or sex × fatigue interactions were found for SD of AP and ML COP positions.

#### 3.3.3. Mean COP sway velocity

**FIGURE 4:**
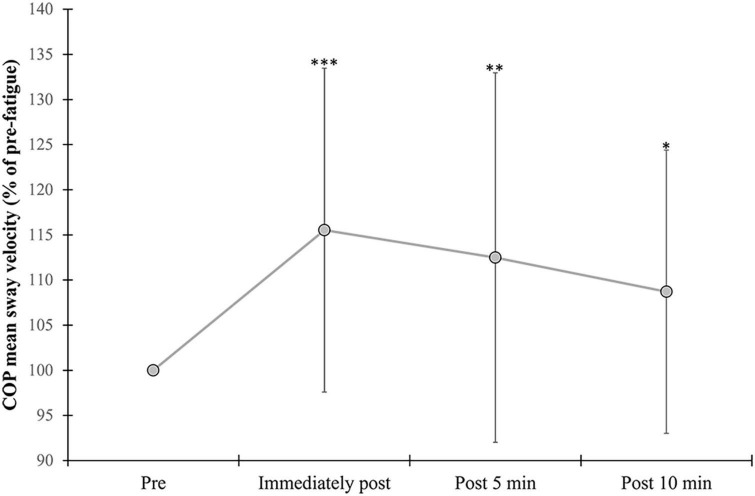
Changes in mean COP sway velocity. Percent change from pre-exercise values are provided immediately after, and at 5 and 10 min following the alternate isometric exercise of ankle plantar and dorsiflexors during standing forward leaning with eyes open (EO) and closed (EC) and overall (EO + EC). The symbols ***, **, and * indicate *p* = <0.001, *p* = <0.01, and *p* = <0.05, respectively for overall changes as there were no significant vision × fatigue interactions. Overall data include standard deviation bars.

A significant effect of vision (*F* = 74.79, *p* = <0.001, η^2^ = 0.721) was found for COP sway velocity, with higher values observed during the EC compared with the EO condition (Table 3). A significant fatigue effect (*F* = 11.16, *p* = <0.001, η^2^ = 0.278) was found due to a significant increase in mean COP sway velocity following exercise compared to pre (immediately: *p* = <0.001, 5 min: *p* = 0.009, and 10 min: *p* = 0.031) (Figure 4). No interactions were found for COP sway velocity.

#### 3.3.4. 95% ellipse of COP sway area

Only a main effect of vision was found for sway area (*F* = 56.37, *p* = <0.001, η^2^ = 0.660), with higher values observed during the EC compared with EO condition (Table 3).

#### 3.3.5. Frequency analysis of COP AP and ML movements for three bands (0–0.3, 0.3–1, and 1–3 Hz)

**FIGURE 5:**
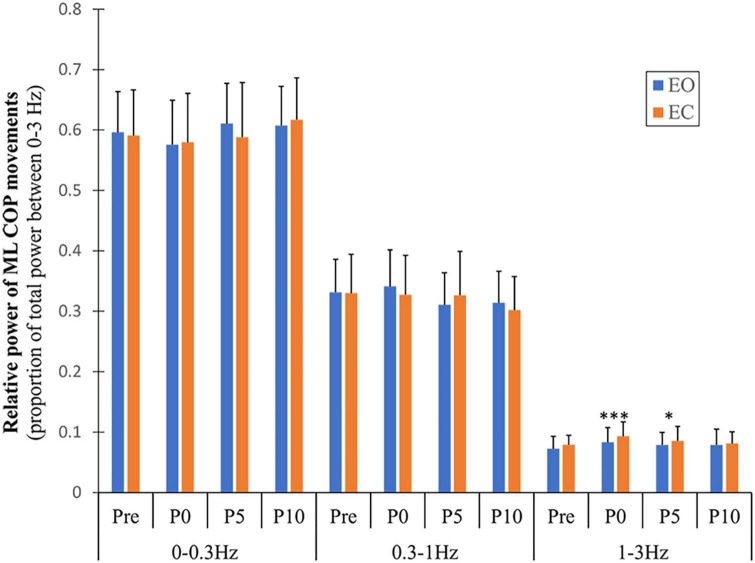
Changes in relative power of ML COP frequency bands with fatigue and recovery. The power spectrum was divided into three frequency bands (0–0.3, 0.3–1, and 1–3 Hz) and expressed as a proportion of total power. Data are shown before (Pre) and immediately (P0), and at 5 and 10 min (P5 and P10) following the alternate isometric exercise of ankle plantar and dorsiflexors for both eyes open (EO) and closed (EC) conditions. The symbols *** and * indicate *p* = <0.001 and *p* = <0.05, respectively for Pre (baseline) to P0 and P5 changes in spectral power of the 1–3 Hz band with EO. Overall data include standard deviation bars.

A significant main effect of vision (*F* = 6.379, *p* = 0.017, η^2^ = 0.180) was found for the ML high frequency band (1–3 Hz), with greater values observed during the EC compared to the EO condition. A main effect of fatigue in the same band (*F* = 3.991, *p* = <0.001, η^2^ = 0.721) was found due to a significant increase in relative power of this band following exercise compared to pre (immediately: *p* = <0.001 and 5 min: *p* = 0.046) (Figure 5). Although the other AP/ML frequency bands showed significant time effects (Supplementary Appendix A), these effects were due to differences between time points during recovery (from immediately to 10 min after exercise), which were not considered fatigue-induced effects in the present study.

### 3.4. SOL H-reflex

**FIGURE 6:**
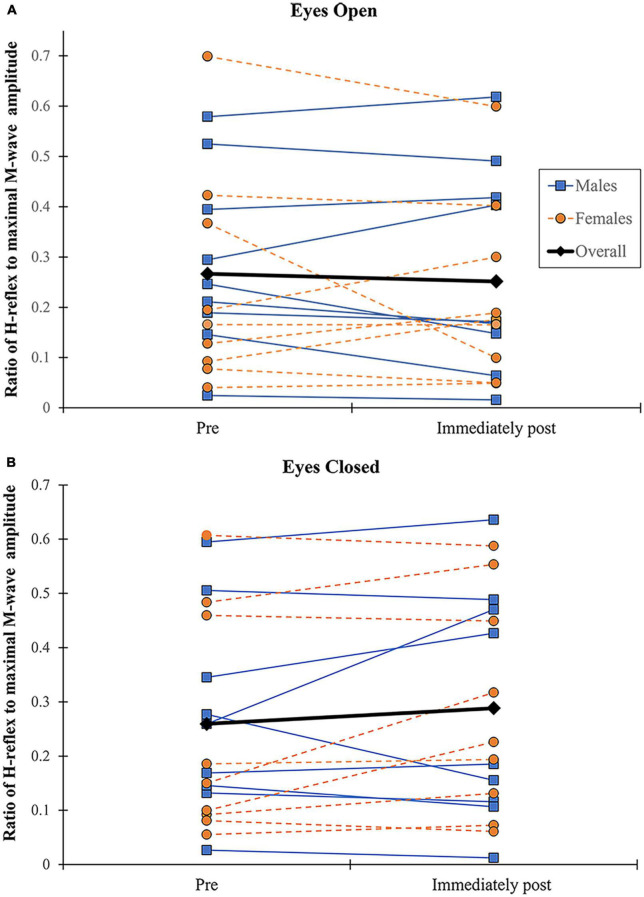
Changes in H/M ratio. H/M ratio shown before and immediately following alternate isometric exercise of ankle plantar and dorsiflexors during standing forward leaning tasks with eyes open **(A)** and closed **(B)**. M (blue) and F (orange) indicate males and females, respectively.

No significant effects were found for the H/M ratio according to vision (*F* = 0.727, *p* = 0.406, η^2^ = 0.043), sex (*F* = 0.727, *p* = 0.406, η^2^ = 0.008), fatigue (*F* = 0.167, *p* = 0.688, η^2^ = 0.010), and their interactions. Participants showed inconsistent changes in the ratio with fatigue regardless of sex and vision conditions (Figure 6). Because the criterion to elicit a comparable H reflex across time points (small M-wave with consistent amplitude) was not met in several participants during recovery (5- and 10-min post-exercise), only data for pre and immediately post-exercise were considered.

### 3.5. Co-contraction index

At pre-fatigue, the mean activity level (% MVIC) of ankle muscles (TA, SOL, and GL) was 4.7, 24.1, and 16.1 during FL-EO, as well as 5.4, 25.8, and 12.3 during FL-EC, respectively. Table 4 shows the mean values and SDs of the co-contraction index at all time points before and after exercise. A significant main effect of vision (*F* = 5.246, *p* = 0.030, η^2^ = 0.163) was found for the TA-SOL co-contraction index, with great values in EC compared to EO throughout all timepoints. No significant effects of sex and fatigue (or their interaction) were found for either of the co-contraction indices (TA-GL and TA-SOL).

**TABLE 4:** Mean values (SD) of co-contraction indices (TA-SOL and TA-GL).

Groups	Co-contraction indices	Mean values (SD)							
		**Eyes open**				**Eyes closed**			
		**Pre**	**Post 0**	**Post 5**	**Post 10**	**Pre**	**Post 0**	**Post 5**	**Post 10**
Overall (*n* = 24)	TA-SOL	3.92 (1.96)	3.98 (1.94)	3.83 (1.89)	4.11 (1.92)	4.68 (3.75)	5.12 (4.14)	4.85 (3.71)	5.43 (3.50)
	TA-GL	4.46 (2.09)	4.62 (2.16)	4.30 (1.92)	4.80 (2.22)	4.82 (3.81)	4.98 (3.45)	5.38 (3.53)	5.45 (3.38)
Males (*n* = 10)	TA-SOL	4.14 (2.25)	4.16 (2.30)	4.11 (1.95)	4.31 (2.58)	5.11 (5.20)	5.603 (4.59)	5.42 (4.15)	6.15 (3.81)
	TA-GL	4.14 (2.47)	4.49 (2.23)	4.13 (2.28)	4.92 (2.28)	5.17 (4.69)	5.27 (5.03)	5.53 (4.80)	5.95 (3.86)
Females (*n* = 14)	TA-SOL	3.71 (1.37)	3.81 (1.68)	3.56 (1.45)	3.91 (1.58)	4.28 (2.71)	4.68 (3.19)	4.31 (2.32)	4.75 (3.09)
	TA-GL	4.75 (1.95)	4.75 (2.09)	4.46 (1.95)	4.70 (1.92)	4.49 (1.87)	4.70 (2.00)	5.24 (2.96)	4.98 (2.97)

These values were assessed before and immediately following fatiguing exercise, and throughout recovery (5 and 10 min). TA-SOL, co-contraction index between tibialis anterior and soleus; TA-GL, co-contraction index between tibialis anterior and gastrocnemius lateralis.

### 3.6. Pearson’s correlation

No significant correlation was found between pre to immediately post-fatigue changes in any COP variables and co-contraction indices (TA-SOL and TA-GL) (Table 5). There was also no significant correlation between the H/M ratio and the preceding 50-ms EMG amplitude of each of GL and SOL at each of pre and immediately post-exercise, and between pre to immediately post-exercise changes in the H/M ratio and the preceding 50-ms EMG amplitude of TA or RPF value (immediately after exercise) during each of FL-EO and FL-EC (Table 6).

**TABLE 5:** Results of Pearson’s correlation analyses.

Vision	COP sway variables	Co-contraction indices					
		**TA-GL**			**TA-SOL**		
		***r***	**95% CI of *r***	***p*-Value**	***r***	**95% CI of *r***	***p*-Value**
EO	ML position	0.115	−0347 to 0.541	0.538	0.169	−0.313 to 0.563	0.362
	AP position	−0.291	−0.521 to 0.064	0.112	−0.317	−0.518 to −0.011	0.082
	ML SD	−0.074	−0.428 to 0.026	0.694	−0.084	−0.471 to 0.196	0.652
	AP SD	−0.290	−0.551 to 0.087	0.113	−0.185	−0.471 to 0.218	0.319
	Sway velocity	−0.114	−0.365 to 0.150	0.541	−0.044	−0.252 to 0.213	0.816
	Sway area	−0.273	−0.604 to −0.022	0.138	−0.195	−0.529 to 0.026	0.292
EC	ML position	−0.016	−0.094 to 0.221	0.930	−0.014	−0.090 to 0.270	0.942
	AP position	0.165	−0.421 to 0.570	0.374	0.319	−0.219 to 0.646	0.081
	ML SD	−0.044	−0.363 to 0.461	0.813	−0.228	−0.497 to 0.146	0.218
	AP SD	0.099	−0.177 to 0.474	0.596	0.115	−0.169 to 0.573	0.406
	Sway velocity	−0.068	−0.320 to 0.289	0.715	−0.190	−0.414 to 0.132	0.306
	Sway area	0.024	−0.298 to 0.505	0.897	−0.056	−0.324 to 0.353	0.766

The correlations were assessed between pre to immediately post-fatigue changes in co-contraction indices (TA-GL and TA-SOL) and COP variables. TA-GL, co-contraction index between tibialis anterior (TA) and gastrocnemius lateralis (GL); TA-SOL, co-contraction index between TA and soleus (SOL); EO, eyes open; EC, eyes closed; ML, mediolateral; AP, anteroposterior; SD, standard deviation; *r*, correlation coefficient; CI, confidence interval.

**TABLE 6:** Results of Pearson’s correlation analysis.

Time period (or point)	Muscle or RPF	H/M ratio					
		**EO**			**EC**		
		***r***	**95% CI of *r***	***p*-Value**	***r***	**95% CI of *r***	***p*-Value**
Pre to P0	TA	−0.015	−0.455 to 0.485	0.952	0.040	−0.307 to 0.428	0.875
Pre	SOL	0.273	−0.161 to 0.643	0.272	0.135	−0.233 to 0.590	0.593
P0	SOL	0.036	−0.300 to 0.308	0.888	0.114	−0.291 to 0.484	0.652
Pre	GL	0.162	−0.121 to 0.585	0.520	0.193	−0.128 to 0.565	0.442
P0	GL	0.018	−0.278 to 0.456	0.942	0.029	−0.332 to 0.385	0.910
P0	RPF	0.109	−0.308 to 0.593	0.689	0.412	0.020 to 0.728	0.113

The correlations are assessed between soleus (SOL) H/M ratio and either ankle muscle activation (for 50 ms prior to the onset of H-reflex) or the rating of perceived fatigue (RPF). H/M ratio, a ratio of soleus H-reflex to maximal M-wave amplitude; EO, eyes open; EC, eyes closed; Pre, before exercise; P0, immediately after exercise; TA, tibialis anterior; GL, gastrocnemius lateralis; *r*, correlation coefficient; CI, confidence interval.

## 4. Discussion

The main aim of this study was to compare postural control changes with ankle muscle fatigue during the FL task between healthy young males and females in different vision conditions (EO vs. EC). The secondary aim was to determine fatigue-induced alterations in SOL motoneuron excitability with activation of Ia afferents (H-reflex) during the FL task according to sex and vision. Our findings showed no significant differences in fatigue-induced COP sway changes (from pre to post exercise) between males and females during the FL task regardless of vision conditions, with no significant fatigue-induced changes in co-contraction indices. This study also found no significant effects of fatigue on the H/M ratio independent of sex and vision conditions. The findings are not consistent with our hypotheses, where we expected males to show greater postural alterations and SOL H-reflex depression with fatigue, and such results would be more pronounced with the withdrawal of vision.

### 4.1. Effects of ankle muscle fatigue on postural control

Fatigue of ankle PF and dorsiflexors led to a significant increase in overall mean COP sway velocity during the FL task up to 10 min following exercise. This is in line with previous findings of unipedal or bipedal quiet standing tasks (Boyas et al., 2011, 2013a,b,c). It has been suggested that such an increase in sway velocity with fatigue may be the consequence of an increase in ankle stiffness to maintain postural stability (Winter et al., 1998). Such stiffening behavior could be caused by alterations in sensitivity of muscle spindles and related sensory reweighting with fatigue, leading to reorganization of multi-joint coordination and altered or compensated stability control via the CNS (Paillard, 2012).

However, we found no significant effect of ankle muscle fatigue on co-contraction indices, as well as no significant correlation between pre- to immediately post-fatigue changes in COP variables and co-contraction indices (Table 5). The present lack of a fatigue effect on co-contraction is surprising given that ankle co-contraction could increase ankle stiffness (resistance that a joint offers through muscles and other soft tissues in response to an applied moment; see Davis and DeLuca, 1996) to maintain static stability (Baratta et al., 1988; Winter et al., 1998; Carpenter et al., 2001). One possible explanation for the absence of a fatigue effect could be due to the requirements of the FL task. The FL position, for example, involves greater activation (% MVIC) in both agonist and antagonist muscles (SOL: 26 %, GL: 12 %, and TA: 5 %) and greater co-contraction index (TA-SOL: 6.5 and TA-GL: 7.8), as compared with quiet standing [SOL: 10% and TA: 2 % (Donath et al., 2016); and TA-SOL: 2.4 (our calculation based on Donath et al., 2016)], which could limit the extent of agonist/antagonist modulation available to increase ankle stiffness to compensate for fatigue. Thus, in the present study, the fatigue-induced increase in sway velocity may instead indicate the use of more corrective actions at the ankle independent of stiffness, as COP sway velocity has been shown to be highly correlated with COM sway acceleration during quiet standing (Masani et al., 2014). The increase in sway velocity may also indicate a potential increase in proximal joint contribution to maintaining postural control during challenging static tasks. For example, Boyas et al. (2013a,b) found that significant increases in ankle and/or hip flexion of the supporting and free legs with fatigue of ankle PF during one-leg standing were accompanied with increases in AP/ML COP sway velocity.

Such a potential compensatory change in proximal joint position, which could occur with insufficient ankle torque needed for optimal control (Horak, 2006), may also have led to the similar performance (amplitude of COP movement: AP/ML position, SD, and sway area) in participants after compared with before the fatiguing exercise. A compensation by other muscle groups, such as ankle mediolateral stabilizers and toe flexors, as suggested in previous studies (Lundin et al., 1993; Harkins et al., 2005; Boyas et al., 2013b), could also be involved. Furthermore, sensory systems likely less or not directly affected by ankle fatigue [e.g., vestibular and neck somatosensory inputs, see Pinsault and Vuillerme (2008)] could play a greater compensatory role for the reduced ability of fatigued ankle PF to maintain FL standing stability with a neutral head position. The analysis of the frequency content of COP movements, however, showed no effect of fatigue for all three frequency bands regardless of vision conditions, except for a slight but significant increase in the ML 1–3 Hz frequency band, potentially reflecting an increased contribution from proprioception with fatigue. The general lack of a fatigue effect on COP frequency content could be due to the characteristics of the FL task. Because the task relied on using a representation of the COP to guide body posture, this may have lessened the possibility for sensory reweighting/compensation with fatigue, especially from the vision system (see section “4.4. Interactions between vision and fatigue on postural control”). Also, for the first block of COP data measured immediately after exercise in the present study, a time delay of approximately 1 min occurred for the transition from the dynamometer to the force platform, as well as for any adjustment to the electrical stimulation intensity. Given this delay, the lack of significant fatigue-induced changes in the COP amplitude variables may indicate that the effect of fatigue can be “short lived.” This is consistent with previous results (Boyas et al., 2013b), where it was observed that COP variables were affected only immediately after a tiptoe standing exercise with no significant time delay between fatigue and testing, but not after a 60-s recovery period.

### 4.2. Sex differences in postural control

We found that males had greater values in SD of overall mean AP COP position, sway area, and ML high frequency band (1–3 Hz), as compared with females. These findings indicate that males may have lesser ability to regulate stability when leaning forward close to the LOS as compared with females. Such sex difference in postural stability could be due to documented advantages in biomechanical factors such as a lower COM caused by a wider pelvic base and a greater valgus position of the legs in females compared with males (Zeller et al., 2003; Era et al., 2006; Gribble et al., 2009; Paillard, 2012). Although body height and weight could be significant contributors to sex difference in postural control during some tasks (Era et al., 2006; Kim et al., 2012), we still observed the sex difference in SD of mean AP position with COP data normalized by height and weight, and also sex differences in SD of mean ML position and sway velocity with the weight-normalized data became appeared with the normalization.

### 4.3. Interactions between sex and fatigue on postural control

No significant sex × fatigue interactions were found for COP sway variables (except for mean ML COP position; however, not reflected in *post-hoc t*-tests) and the co-contraction indices, indicating generally similar postural control changes with fatigue between males and females. This is consistent with previous findings, which showed no significant sex differences in COP sway changes with fatigue of the ankle or hip muscles during unipedal standing tasks (Nelson and Johnson, 1973; McMullen et al., 2011). This is not necessarily surprising given similar performance fatigability (the time to task failure) and perceived fatigability (increase in RPF values following exercise, see Figure 3) between males and females (McMullen et al., 2011).

The similar fatigability in males and females may be dependent on the specific exercise/task used to induce fatigue. It has often been reported that females can be less fatigable than males during sustained isometric exercise (Hunter, 2014, 2016), mainly due to their typical lesser muscle mass and strength [limiting blood flow occlusion and accumulation of metabolites, as suggested in Miller et al. (1993), Hunter and Enoka (2001), and Kavanagh et al. (2020)]. The extent of sex differences in performance fatigability during sustained isometric exercise of upper and lower limbs tends to be greater for low-intensity contractions (e.g., 20% MVIC) compared to moderate to high intensity contractions (e.g., 50–80%) (Maughan et al., 1986; Yoon et al., 2007). This may be due to the greater extent of sex differences in muscle perfusion (females > males) during such low-intensity exercise compared to moderate to high intensity in the context of sustained isometric contraction (Hunter, 2014, 2016). In contrast to sustained low-intensity isometric exercise, the maximal intermittent isometric exercise used in the present study may not have favored the emergence of sex differences in muscle perfusion during the exercise (Hunter, 2014, 2016) and consequently in ankle muscle fatigue and ultimately in fatigue-induced postural changes.

Furthermore, the findings of a systematic review summarizing 46 studies of various fatigued muscle groups (Hunter, 2016) suggest that ankle plantar and dorsiflexors show lesser sex differences in fatigability as compared with other muscle groups. This could be explained by smaller sex differences in relative area of type I (fatigue resistant) muscle fibers and muscle mass and/or strength for the ankle muscles compared with other muscle groups (Coggan et al., 1992; Miller et al., 1993; Staron et al., 2000; Larsson et al., 2006), which would contribute to the similar fatigability between males and females directly and through muscle metabolism (which can affect the accumulation of metabolites and motor neuron activation) (Hunter, 2014, 2016). Again, the absence of sex differences in fatigue-induced postural changes in the present study could thus be attributed to similar ankle muscle fatiguability between the sex groups.

### 4.4. Interactions between vision and fatigue on postural control

Several studies looking at fatigue of ankle PF have found greater postural sway changes (or impairment) with no-vision and/or disturbed vision compared to normal vision during upright standing on one leg (Bisson et al., 2010; Soleimanifar et al., 2012; Boyas et al., 2013a; Barbieri et al., 2019) or two legs (Vuillerme et al., 2001, 2006), suggesting a significant role of visual information on compensating declines in the neuromuscular system and proprioception of fatigued ankle PF to maintain stability (Paillard, 2012). However, we found no significant vision × fatigue interaction for any of the COP sway variables, as well as for both co-contraction indices, indicating that the withdrawal of vision did not significantly affect fatigue-induced postural control changes. These results are in accordance with a few studies investigating ankle PF fatigue.

For example, two previous studies (Vuillerme et al., 2006; Boyas et al., 2013b) found that the removal of vision does not increase postural sway during one or two-leg standing following sustained or repeated tiptoes until task failure. These authors suggested that the presence of a significant level of fatigue may have restrained vision from compensating fatigue-induced postural changes (Boyas et al., 2013b), or the distance of the visual target used (4 m) may have eliminated the compensatory effect of vision on postural control changes with fatigue (which was different with a 1-m visual target leading to significant vision effects) (Vuillerme et al., 2006). Even though we used a relatively close visual “target” distance (1.8 m), the specific characteristics of the postural task used in the present study, in contrast to a simple quiet standing task, may have lessen the potential effect of vision to compensate for fatigue-related alterations in postural control. The use of the representation of the COP on a computer screen to accomplish the FL task involved participants initially reaching the target (70% anterior LOS) before closing their eyes and thus likely memorizing the position where the COP position was to be maintained in the absence of vision. This may have reduced the potential effect of vision and would be different from the use of vision in the context of a quiet standing task while fixating a visual target. Lastly, compensatory proximal joint control could be prioritized rather than ankle control to maintain stability when removing vision in a state of ankle fatigue, which may have led to the lack of a vision effect. For example, Boyas et al. (2013b) found that a backward movement of the pelvis (hip flexion) produced by fatigue of ankle PF during one-leg standing task significantly increased with EC (15%) compared to EO, while observing a similar fatigue-induced increase in AP/ML COP sway velocity independent of vision conditions (EO vs. EC).

We did find a significant sex × time × vision interaction for mean ML sway position, where only males showed a left shift during recovery (immediately to 5 min after exercise) and only with EC. This may indicate a different postural control strategy with fatigue and recovery between males and females under certain conditions. However, we interpret this result with caution as none of the post-exercise values were different from pre-exercise.

### 4.5. Motoneuron excitability from Ia afferent (proprioceptive) input at the ankle

No significant effects were found for the H/M ratio according to all independent variables, as there was no consistent pattern of changes in the ratio with fatigue regardless of sex and vision conditions (Figure 6). This is consistent with previous findings by Laudani et al. (2009), where different changes (increase or decrease) in H-reflex amplitude during quiet standing were found across healthy young adults immediately following an initial set of repetitive tiptoe movements (out of five sets).

The lack of a fatigue effect on the H/M ratio may be attributed to different changes (increase or decrease) in presynaptic inhibition with ankle fatigue among the participants, with varying involvement of descending command modulation and/or muscle metabolic changes. In the present study, an increase in the H/M ratio with fatigue was noted in participants in seven EO and nine EC conditions. In those instances, fatiguing exercise could have led to descending inhibitory input onto the interneurons accountable for pre-synaptic inhibition of Ia afferents, causing an enhancement of the stretch reflex efficacy at the spinal level (Trimble and Harp, 1998). In contrast, a decrease in the H/M ratio with ankle fatigue in the other participants/conditions (11 EO and 9 EC) could be attributed to a potential increase in pre-synaptic inhibition of Ia afferents, possibly due to facilitation of small-diameter group III and IV afferents under the influence of muscle metabolic changes (e.g., reduced oxygen availability and accumulation of metabolites) (Kaufman et al., 1983; Duchateau and Hainaut, 1993; Sinoway et al., 1993). Such depression in the H/M ratio could also have been caused by reciprocal inhibition due to increased activation of antagonists (TA) with fatigue (Ritzmann et al., 2016). However, we did not observe a significant association between pre-to-immediately post-fatigue changes in the H/M ratio and RPF value [which changes can be related to the metabolism within muscles (Borg, 1982; Williams, 2017)] or TA activation (taken immediately before the onset of SOL H-reflex).

Other than the neurophysiological factors mentioned above, psychological factors associated with the experimental conditions could contribute to the variability in the H/M ratio (Chen and Zhou, 2011). For example, anxiety imposed by a threat to postural stability (see Sibley et al., 2007) could have played a role, especially during the FL-EC condition. Intrinsic forward or backward sway during the FL task at the moment when the SOL H-reflex is elicited may be another potential confounding factor. An increase in SOL H-reflex with intrinsic anterior sway during quiet standing has been reported (Tokuno et al., 2007). We, however, found no significant correlation between the H/M ratio and each of SOL and GL activation (50 ms before the onset of SOL H-reflex), which is reported to be highly correlated with AP COP position (Tokuno et al., 2007). The inconsistent changes in SOL H-reflex in the present study may thus be due to different neurophysiological and psychological conditions among the participants during their experimental session.

A few limitations should be mentioned for the present study. First, movements and positions of proximal joints/segments (e.g., hip and trunk movements) that could compensate for ankle muscle fatigue to maintain stability were not assessed. Second, the true relation between SOL H-reflex and COP data remains uncertain in the present study because COP data and SOL reflex were not assessed during the same FL task. Third, EMG data for the co-contraction index were not assessed bilaterally, which might have prevented us from detecting asymmetrical effects of fatigue on the index. However, we think that the bilateral nature of the fatigue task and the AP focus of the FL task would minimize such asymmetrical effect. The EMG data were also not normalized to a maximum value, which might have affected between-subject effects (a main effect of sex) on the index. However, significant relative changes of the index (within-subject effects over time or across vision conditions) would likely not be affected by the lack of normalization. Fourth, there were no MVIC measurements during recovery due to the time limit to measure H-reflex and COP data, which was limited to determine the true effects of muscle (or neuromuscular) fatigue on standing stability. Fifth, as previously mentioned, the approximate 1-min time delay after the fatiguing exercise needed to transition to the force platform and for stimulation intensity adjustment, may have lessened a significant fatigue effect on FL task control. We have recently shown a relatively greater fatigue effect on COP movements during the same FL task with a protocol requiring only a 30 s transition after exercise (see Jo et al., 2022). The lesser fatigue effect may also be partly explained by the number of bouts of fatiguing exercise performed, where only a single set was performed in the present study in contrast to four bouts in our previous study (Jo et al., 2022). However, this should be interpreted carefully, as perceived fatigability after exercise was significant and prolonged in the present study. Lastly, we did not obtain information on the phases of the menstrual cycle in female participants, which has been found to affect postural stability (Lee and Yim, 2016; Khowailed and Lee, 2021). However, this would likely not be a major confounding factor to sex differences in postural control changes with fatigue, given the smaller effect of the menstrual cycle on fatigability compared to other general sex differences in fatigability (Hunter, 2016).

## 5. Conclusion

We found similar fatigue-induced postural control changes between males and females during the FL task regardless of vision conditions, where both sex groups had a significant increase in sway velocity following fatiguing exercise of ankle muscles, with no significant changes in ankle co-contraction. Also, no fatigue effect was found on SOL H-reflex, independent of sex and vision conditions (EO vs. EC). The lack of sex differences on postural control and H-reflex with fatigue is not surprising given similar performance and perceived fatigability between the sex groups in the present study. Our findings highlight the importance of considering (fatiguing and postural) task specificity, the muscle group studied, as well as individual characteristics when studying sex differences related to the effects of fatigue on postural control.

## Data availability statement

The raw data supporting the conclusions of this article will be made available by the authors, without undue reservation.

## Ethics statement

The studies involving human participants were reviewed and approved by the University of Ottawa Research Ethics Board (# H11-14-23; University of Ottawa) and Bruyère Continuing Care Research Ethics Board (#M16-08-038; Bruyère Research Institute). The patients/participants provided their written informed consent to participate in this study.

## Author contributions

DJ and MB conceptualized the study. DJ carried out the data collection and statistical analyses, and wrote and edited the manuscript. MB participated in all research aspects of the study and provided advice and content expertise, and reviewed and edited the manuscript. Both authors contributed to the article and approved the submitted version.
