# Supplementary material for: Sex differences concerning the effects of ankle muscle fatigue on static postural control and spinal proprioceptive input at the ankle
Source: Front Hum Neurosci. 2023 Jul 5;17:1015597. doi: 10.3389/fnhum.2023.1015597 (PMC10355328; doi:10.3389/fnhum.2023.1015597)
Supplement: Supplementary file 1 [file Table_1.DOCX]

**APPENDICES**

**Appendix A.** Summary of statistical results (ANOVAs) of changes in center of pressure (COP) sway parameters, frequency analysis of COP movements, co-contraction index (TA-SOL and TA-GL), soleus (SOL) H-reflex amplitude, and the Borg rating of perceived fatigue (RPF) during standing forward leaning (FL) task according to vision, sex, and time (including fatigue). TA-SOL = between tibialis anterior and soleus; TA-GL = between tibialis anterior and gastrocnemius lateralis.

| Effects | COP sway parameters | | | | | | | | | | | | | | | | | |
| --- | --- | --- | --- | --- | --- | --- | --- | --- | --- | --- | --- | --- | --- | --- | --- | --- | --- | --- |
|  | *AP position* | | | *AP SD* | | | *ML position* | | | *ML SD* | | | *Velocity* | | | *Area* | | |
|  | *F* | *P* | *ηp2* | *F* | *P* | *ηp2* | *F* | *P* | *ηp2* | *F* | *P* | *ηp2* | *F* | *P* | *ηp2* | *F* | *P* | *ηp2* |
| *Vision* | 16.04 | **<0.001** | 0.356 | 114.0 | **<0.001** | 0.797 | 3.352 | 0.077 | 0.104 | 120.2 | **<0.001** | 0.806 | 74.79 | **<0.001** | 0.721 | 56.37 | **<0.001** | 0.660 |
| *Sex* | 2.015 | 0.166 | 0.065 | 4.334 | **0.046** | 0.130 | 1.715 | 0.201 | 0.056 | 3.215 | 0.083 | 0.100 | 1.611 | 0.214 | 0.053 | 3.432 | 0.074 | 0.106 |
| *Time* | 2.169 | 0.097 | 0.070 | 1.035 | 0.381 | 0.034 | 1.871 | 0.140 | 0.061 | 0.317 | 0.751 | 0.011 | 11.16 | **<0.001** | 0.278 | 1.124 | 0.338 | 0.037 |
| *Time X Vision* | 1.740 | 0.165 | 0.057 | 0.794 | 0.500 | 0.027 | 1.567 | 0.203 | 0.051 | 0.278 | 0.787 | 0.009 | 2.529 | 0.063 | 0.080 | 0.831 | 0.461 | 0.028 |
| *Time X Sex* | 0.750 | 0.525 | 0.025 | 0.815 | 0.489 | 0.027 | 3.331 | **0.023** | 0.103 | 1.451 | 0.241 | 0.048 | 1.004 | 0.395 | 0.033 | 1.593 | 0.206 | 0.052 |
| *Time X Vision X Sex* | 0.724 | 0.540 | 0.024 | 1.217 | 0.308 | 0.040 | 3.293 | **0.024** | 0.102 | 2.403 | 0.091 | 0.077 | 1.107 | 0.351 | 0.037 | 2.016 | 0.131 | 0.065 |

| Effects | Frequency analysis of AP and ML COP movements | | | | | | | | | | | | | | | | | |
| --- | --- | --- | --- | --- | --- | --- | --- | --- | --- | --- | --- | --- | --- | --- | --- | --- | --- | --- |
|  | *Power in three frequency bands in an AP direction (% total power)* | | | | | | | | | *Power in three frequency bands in an ML direction (% total power)* | | | | | | | | |
|  | *0-0.3 Hz* | | | *0.3-1 Hz* | | | *1-3Hz* | | | *0-0.3 Hz* | | | *0.3-1 Hz* | | | *1-3Hz* | | |
|  | *F* | *P* | *ηp2* | *F* | *P* | *ηp2* | *F* | *P* | *ηp2* | *F* | *P* | *ηp2* | *F* | *P* | *ηp2* | *F* | *P* | *ηp2* |
| *Vision* | 2.624 | 0.116 | 0.083 | 3.380 | 0.076 | 0.104 | 3.052 | 0.091 | 0.095 | 0.091 | 0.765 | 0.003 | 0.117 | 0.735 | 0.004 | 6.379 | **0.017** | 0.180 |
| *Sex* | 0.125 | 0.727 | 0.004 | 0.004 | 0.951 | <0.001 | 0.221 | 0.642 | 0.008 | 0.978 | 0.331 | 0.033 | 0.888 | 0.354 | 0.030 | 0.461 | 0.503 | 0.016 |
| *Time* | 3.854 | **0.015** | 0.117 | 4.299 | **0.010** | 0.129 | 2.616 | 0.066 | 0.083 | 3.991 | **0.016** | 0.121 | 3.941 | **0.015** | 0.120 | 5.979 | **0.001** | 0.171 |
| *Time X Vision* | 2.069 | 0.117 | 0.067 | 2.453 | 0.077 | 0.078 | 1.260 | 0.293 | 0.042 | 1.332 | 0.270 | 0.044 | 1.179 | 0.322 | 0.039 | 0.996 | 0.394 | 0.033 |
| *Time X Sex* | 1.090 | 0.355 | 0.036 | 1.284 | 0.286 | 0.042 | 1.784 | 0.165 | 0.058 | 0.489 | 0.657 | 0.017 | 0.485 | 0.667 | 0.016 | 0.773 | 0.504 | 0.026 |
| *Time X Vision X Sex* | 0.425 | 0.717 | 0.014 | 0.584 | 0.606 | 0.020 | 0.672 | 0.551 | 0.023 | 0.192 | 0.895 | 0.007 | 0.165 | 0.911 | 0.006 | 2.309 | 0.089 | 0.074 |

| Effects | Co-contraction index | | | | | | SOL-H reflex | | | RPF | | |
| --- | --- | --- | --- | --- | --- | --- | --- | --- | --- | --- | --- | --- |
|  | *TA-GL* | | | *TA-SOL* | | |  |  |  |  |  |  |
|  | *F* | *P* | *ηp2* | *F* | *P* | *ηp2* | *F* | *P* | *ηp2* | *F* | *P* | *ηp2* |
| *Vision* | 2.488 | 0,126 | 0.084 | 5.246 | **0.030** | 0.163 | 0.727 | 0.406 | 0.043 | n/a | n/a | n/a |
| *Sex* | 0.023 | 0.880 | 0.001 | 0.560 | 0.461 | 0.020 | 0.122 | 0.732 | 0.008 | 0.013 | 0.912 | 0.001 |
| *Time* | 1.128 | 0.330 | 0.040 | 1.626 | 0.190 | 0.057 | 0.176 | 0.688 | 0.010 | 41.37 | **<0.001** | 0.633 |
| *Time X Vision* | 1.466 | 0.234 | 0.051 | 0.967 | 0.413 | 0.035 | 3.872 | 0.067 | 0.195 | n/a | n/a | n/a |
| *Time X Sex* | 0.563 | 0.570 | 0.020 | 0.161 | 0.922 | 0.006 | 0.098 | 0.759 | 0.006 | 0.591 | 0.545 | 0.024 |
| *Time X Vision X Sex* | 0.444 | 0.702 | 0.016 | 0.224 | 0.879 | 0.008 | 0.492 | 0.493 | 0.030 | n/a | n/a | n/a |

**Appendix B.** Summary of statistical results (ANOVAs) of changes in center of pressure (COP) sway parameters (normalized by height and weight during standing forward leaning (FL) task according to vision, sex, and fatigue (time).

| Effects | COP sway parameters (normalized by height) | | | | | | | | | | | | | | | | | |
| --- | --- | --- | --- | --- | --- | --- | --- | --- | --- | --- | --- | --- | --- | --- | --- | --- | --- | --- |
|  | *AP position* | | | *AP SD* | | | *ML position* | | | *ML SD* | | | *Velocity* | | | *Area* | | |
|  | *F* | *P* | *ηp2* | *F* | *P* | *ηp2* | *F* | *P* | *ηp2* | *F* | *P* | *ηp2* | *F* | *P* | *ηp2* | *F* | *P* | *ηp2* |
| *Vision* | 17.66 | **<0.001** | 0.378 | 106.1 | **<0.001** | 0.785 | 3.257 | 0.082 | 0.101 | 110.9 | **<0.001** | 0.793 | 68.99 | **<0.001** | 0.704 | 51.83 | **<0.001** | 0.641 |
| *Sex* | 0.006 | 0.937 | <0.001 | 6.968 | **0.013** | 0.194 | 1.668 | 0.207 | 0.054 | 6.160 | 0.019 | 0.175 | 3.435 | 0.074 | 0.106 | 4.358 | 0.046 | 0.131 |
| *Time* | 2.111 | 0.105 | 0.068 | 1.071 | 0.366 | 0.036 | 1.976 | 0.124 | 0.064 | 0.383 | 0.703 | 0.013 | 11.23 | **<0.001** | 0.279 | 1.215 | 0.307 | 0.040 |
| *Time X Vision* | 1.648 | 0.201 | 0.054 | 0.805 | 0.494 | 0.027 | 1.625 | 0.189 | 0.053 | 0.257 | 0.803 | 0.009 | 2.418 | 0.072 | 0.077 | 0.899 | 0.429 | 0.030 |
| *Time X Sex* | 0.764 | 0.518 | 0.026 | 0.885 | 0.452 | 0.030 | 3.391 | **0.022** | 0.015 | 1.463 | 0.238 | 0.048 | 1.187 | 0.319 | 0.039 | 1.654 | 0.194 | 0.054 |
| *Time X Vision X Sex* | 0.706 | 0.500 | 0.024 | 1.254 | 0.295 | 0.041 | 3.324 | **0.023** | 0.103 | 2.363 | 0.095 | 0.075 | 1.060 | 0.371 | 0.035 | 2.047 | 0.126 | 0.066 |

| Effects | COP sway parameters (normalized by weight) | | | | | | | | | | | | | | | | | |
| --- | --- | --- | --- | --- | --- | --- | --- | --- | --- | --- | --- | --- | --- | --- | --- | --- | --- | --- |
|  | *AP position* | | | *AP SD* | | | *ML position* | | | *ML SD* | | | *Velocity* | | | *Area* | | |
|  | *F* | *P* | *ηp2* | *F* | *P* | *ηp2* | *F* | *P* | *ηp2* | *F* | *P* | *ηp2* | *F* | *P* | *ηp2* | *F* | *P* | *ηp2* |
| *Vision* | 19.94 | **<0.001** | 0.407 | 88.42 | **<0.001** | 0.753 | 3.354 | 0.077 | 0.104 | 84.55 | **<0.001** | 0.745 | 56.61 | **<0.001** | 0.661 | 43.76 | **<0.001** | 0.601 |
| *Sex* | 1.510 | 0.229 | 0.049 | 8.937 | **0.006** | 0.236 | 1.522 | 0.227 | 0.050 | 8.547 | **0.007** | 0.228 | 5.041 | **0.033** | 0.148 | 4.984 | 0.033 | 0.147 |
| *Time* | 2.367 | 0.076 | 0.075 | 1.290 | 0.283 | 0.043 | 2.078 | 0.109 | 0.067 | 0.634 | 0.536 | 0.021 | 11.13 | **<0.001** | 0.277 | 1.420 | 0.248 | 0.047 |
| *Time X Vision* | 1.955 | 0.147 | 0.063 | 0.777 | 0.510 | 0.026 | 1.538 | 0.210 | 0.050 | 0.372 | 0.714 | 0.013 | 2.582 | 0.059 | 0.082 | 1.019 | 0.377 | 0.034 |
| *Time X Sex* | 0.758 | 0.520 | 0.025 | 1.130 | 0.341 | 0.038 | 3.204 | **0.035** | 0.099 | 1.304 | 0.279 | 0.043 | 1.399 | 0.249 | 0.049 | 1.616 | 0.202 | 0.053 |
| *Time X Vision X Sex* | 0.766 | 0.479 | 0.026 | 1.320 | 0.273 | 0.044 | 3.197 | **0.027** | 0.099 | 1.989 | 0.140 | 0.064 | 0.873 | 0.458 | 0.029 | 1.821 | 0.162 | 0.059 |
